# Sex‐specific differences in the association between regional adiposity, cognition, and brain pathology in older adults with Type II Diabetes

**DOI:** 10.1002/alz.091659

**Published:** 2025-01-09

**Authors:** Ethel Boccara, Sapir Golan, Ithamar Ganmore, Ramit Ravona‐Springer, Abigail Livny, Mery Ben‐Meir, Yossi Azuri, Aron Weller, Michal S Beeri

**Affiliations:** ^1^ The Joseph Sagol Neuroscience Center, Sheba Medical Center, Tel Hashomer Israel; ^2^ Department of Psychology, Bar Ilan University, Ramat Gan Israel; ^3^ Sackler Faculty of Medicine, Tel Aviv University, Tel Aviv Israel; ^4^ Memory clinic, Sheba Medical Center, Tel Hashomer Israel; ^5^ Department of Diagnostic Imaging, Sheba Medical Center, Tel Hashomer Israel; ^6^ Maccabi Health Services, Tel Aviv Israel; ^7^ Gonda Brain Research Center, Bar Ilan University, Ramat Gan Israel; ^8^ Herbert and Jackeline Krieger Klein Alzheimer’s Research Center, Rutgers Biomedical and Health Sciences, Newark, NJ USA

## Abstract

**Background:**

While body mass index (BMI) is widely used to gauge overall adiposity, its accuracy in older age has yielded inconsistent findings. Moreover, BMI does not account for variations in regional fat distribution, which may differ between sexes. This study aims to investigate whether regional adiposity plays a distinct role in impacting cognition and the volumes of AD‐related brain regions in older adults with T2D enrolled in the Israel Diabetes and Cognitive Decline (IDCD) study.

**Methods:**

Regional adiposity was measured by abdominal MRI in 103 participants (33.3% women), including hepatic and pancreatic fat, visceral adipose tissue (VAT) and subcutaneous adipose tissue (SAT). Participants underwent neuropsychological assessment including global cognition, episodic memory (EP), working memory (WM), executive function (EF) and language. A subsample of 52 participants (36% women), underwent brain MRI assessing hippocampus, inferior, middle, and superior frontal gyrus [IFG, MFG, and SFG, respectively]. Partial correlations examined associations of regional adiposity with cognition and brain volumes adjusting for age, sex, education, BMI (and intracranial volume for volumetric MRI).

**Results:**

In the whole sample, higher pancreatic fat approached significance with lower global cognition (r = ‐286, p = 0.054). No associations were found for specific cognitive domains. In men, but not in women, high pancreatic fat was associated with lower global cognition (Men: r = ‐0.336; p = 0.042; Women: r = ‐0.299; p = 0.565), and lower episodic memory (Men: r = ‐0.345; p = 0.036; Women: r = ‐0.064; p = 0.787). In women, but not in men, high hepatic fat was associated with lower working memory (Men: r = 0.035; p = 0.835; Women: r = ‐0.486; p = 0.022). In the whole sample, no associations were found between regional adiposity and brain volumes. Whereas, in men, higher hepatic fat was associated with smaller MFG (Men: r = ‐0.492, p = 0.013; Women: r = 0.021, p = 0.949) and with smaller SFG (Men: r = ‐0.407, p = 0.043; Women: r = ‐0.097, p = 0.765).

**Conclusion:**

In older adults with T2D, who are at high ADRD risk, distinct regional fat depots have different associations with brain and cognition in men and women. Those differential sex effects are consistent with those of our recent publication on middle‐aged high ADRD risk sample. These findings highlight the need to account for sex differences when investigating the role of adiposity in brain aging.

